# Risk identification and assessment of Internet public opinion on public emergencies based on Bayesian network and association rule mining

**DOI:** 10.3389/fpubh.2025.1642960

**Published:** 2025-07-28

**Authors:** Mengjie You, Xuwei Pan, Chuwen Zhu

**Affiliations:** School of Economics Management, Zhejiang Sci-Tech University, Hangzhou, China

**Keywords:** public emergencies, Internet public opinion, risk identification and assessment, BN, association rule mining

## Abstract

Global public emergencies occur frequently, and the risk of Internet public opinion crises in such contexts is gradually increasing. In the dual context of risk society and network society, effectively identifying and assessing Internet public opinion risks on public emergencies poses challenges to the efficiency and response speed of public crisis management. This paper innovatively proposes an Internet public opinion risk identification and assessment method for public emergencies, integrating association rule mining with Bayesian network (BN). The core innovation lies in designing an improved scheme based on the CBA (Classification Based on Associations) algorithm to overcome the limitation of traditional association rule mining in handling non-Boolean data, thereby effectively extracting the correlations among public opinion risk factors to optimize the topological structure of the BN. Building upon this foundation, we construct a BN model with strong interpretability to identify the public opinion key risk factors and key risk chains of different risk levels, as well as to evaluate the public opinion situation. Empirical results demonstrate that, compared with the traditional BN assessment model based on expert experience, the BN model incorporating association rules achieves a 14.4% increase in assessment accuracy and exhibits more pronounced advantages in performance metrics such as precision, recall, specificity, and F-measure. The proposal of this innovative method not only enhances the accuracy of public opinion risk assessment but also provides a new perspective for data-driven identification of key risk factors and research on their complex interactions. Furthermore, it provides an interpretable and computationally efficient decision support tool for public opinion crisis management.

## 1 Introduction

With the profound transformations and multidimensional developments in modern society, the frequency of public emergencies has exhibited an accelerating growth trend. Furthermore, the widespread use of the Internet and the emergence of new media have inclined people to express their thoughts and attitudes toward emergencies online (1, 2). Internet public opinion is the sum of people’s views, attitudes, and emotions about public emergencies (3). Its development trend can, to some extent, exert significant influence on the evolution and changes of real-world emergencies. If negative Internet public opinion spreads uncontrollably, it can easily induce feelings of helplessness and panic among the public, posing a potential threat to social stability and development, and may even undermine government credibility (4). Without timely guidance and appropriate handling, it is likely to trigger new Internet public opinion crises or even offline secondary group events. Therefore, preventing and resolving the risks of Internet public opinion driven by emergencies is an issue that requires urgent attention and response in the dual era of the network society and the risk society. According to the accident causation theory (5), if hazardous sources of accidents are effectively controlled, major risks can be suppressed at their source, thereby preventing accidents from occurring. Therefore, applying modern information technology to thoroughly explore key risk factors of public opinion and their complex interactions, as well as to assess risk level, is of great significance for preventing and mitigating Internet public opinion crises during public emergencies.

The identification and assessment of Internet public opinion risks constitute a crucial topic within the realm of public opinion risk management (6, 7). Although some scholars have established evaluation index systems and assessment methods of public opinion risks, there are still certain limitations. For instance, existing research primarily focused on theoretically constructing evaluation index systems and developing experience-based assessment methods, both of which have a certain degree of subjectivity. There is relatively little research on studying past public opinion risk cases and analyzing causal relationships between risk factors. This neglects the correlation and interweaving nature of indicators, making objective assessment of public opinion risks difficult. Although an increasing number of scholars have begun to utilize modern information technologies such as machine learning to assess and issue warnings for the crisis levels of public opinion, most of them aim at outputting warning levels. There is a scarcity of in-depth research into the causes of different levels of Internet public opinion risk, which hampers the ability to quickly identify the crucial issues based on risk assessment results. BN possesses significant advantages in expressing uncertainty and causal reasoning, and has been applied in research on public opinion situation assessment (8). However, most studies relied on expert opinions or relevant known information to subjectively design BN topology structures. The rationality of network structure is closely related to the effectiveness of the final BN evaluation model (9). Association rule mining can discover the associative characteristics among uncertain factors causing accidents from extensive accident data, so as to identify the causal relationships between these factors. CBA belongs to the extended application of association rule mining algorithms. Through the generation of association rules and classification, it can achieve classification and judgment of non-boolean discrete data. Therefore, compared with the traditional Apriori, the CBA algorithm can be applied to the association rule mining of non-Boolean datasets, making it suitable for the in-depth analysis of causal relationships among risk factors of public opinion in public emergencies. The cases of Internet public opinion in emergencies hold immense value in extracting key risk factors and their causal pathways that may trigger public opinion crises. Currently, there is a lack of mining of network-based intelligence knowledge on emergencies, and a dearth of collaborative research that, after researchers learn prior case knowledge, conducts risk factor identification and risk situation assessment.

Addressing the aforementioned issues, this study, based on the collection of case data on Internet public opinion of public emergencies, combines association rule mining with BN to identify and assess Internet public opinion risks. The study aims to explore the key risk factors and risk pathways that influence the evolution of Internet public opinion risks, and simultaneously conduct early warning assessments of public opinion situations. The research findings offer a theoretical foundation for targeted joint defense and control of Internet public opinion risks associated with public emergencies, cutting off the transmission path of risks.

The contributions of this paper are as follows: (1) A novel method for identifying and assessing risk of Internet public opinion of public emergencies is proposed, which combines association rule mining with BN. (2) Based on the strong association rules among Internet public opinion risk factors, optimize the topological structure of BN to reduce the inherent subjectivity and limitations of expert modeling. (3) It improves the accuracy of Internet public opinion risk assessment. At the same time, it identifies the key risk factors and key risk chains of different risk levels, providing a new perspective for data-driven research on the complex causal mechanisms of Internet public opinion risks. (4) The application scope of the association rule mining method is broadened, offering a reference for future scholars studying the Internet public opinion risks.

The remainder of this paper is structured as follows. Section 2 reviews related work. Section 3 builds the method for identifying and assessing Internet public opinion risks. Section 4, we analyze the experimental results. Sections 5 and 6 present the discussions, conclusions, and future research directions.

## 2 Literature review

### 2.1 Internet public opinion management

The origin of Internet public opinion research can be traced back to the 1990s, when studies in the sociology of the Internet emerged abroad, laying the foundation for the research on Internet public opinion. Subsequently, Internet public opinion gradually became a hot topic and attracted the attention and research of an increasing number of scholars. During the early stages of Internet popularization (1990–2005), research on Internet public opinion primarily focused on its concepts, characteristics, and societal impacts (10). With the rise of social media (2005–2015), research on Internet public opinion shifted to focus on user behavior and content characteristics on social media platforms, further exploring the formation mechanisms, evolutionary patterns, as well as methods for predicting and responding to public opinion events (11–15). Entering the era of big data and artificial intelligence (2015-present), research on public opinion has also entered into a new stage. The monitoring, guidance, and control of network information ecological elements such as topics, sentiments, and popularity have increasingly received attention (16–20). More and more organizations and government agencies have begun to realize that risk management and control of Internet public opinion is becoming an important component of modern social governance. Therefore, building on existing Internet public opinion research, researchers are further exploring its potential risks and impacts through big data mining, natural language processing, machine learning etc., aiming to enhance the public opinion risk management capability of organizations and government agencies (21–25).

In recent years, the pre-control and management of Internet public opinion risk of emergencies is both a research hotspot and a research challenge. Risk assessment is a crucial component of the Internet public opinion risk precontrol system. Most existing studies adopt the approach of constructing “risk assessment index system + assessment model” to evaluate and grade public opinion risks on emergencies (26–30). For instance, Tian et al. (31) employed the 1–9 scale method to obtain indicator weights based on ANP (Analytic Network Process), and then combined Stochastic Petri Net with Markov Chain for quantitative analysis to achieve the purpose of early warning. Wu et al. (32) established a comprehensive assessment index system for public opinion based on the lifecycle theory, then proposed the entropy weight method to calculate index weights and constructed a fuzzy comprehensive evaluation model for Internet public opinion on this basis. Liu et al. (33) introduced a novel risk grading model for Internet public opinion in public health emergencies, which integrates the Analytic Hierarchy Process Sort II (AHPSort II) with the Swing Weighting (SW) method. However, the aforementioned mostly rely on human experience to construct evaluation index systems and assessment methods, which has a certain subjectivity and ignores the correlation and interweaving between indicators, making it difficult to obtain a more objective assessment. With the continuous improvement of monitoring technologies, it has become possible to dynamically obtain various objective indicator data. Machine learning methods such as neural networks and support vector machines, together with optimization algorithm like genetic algorithms, have achieved considerable results in the application of Internet public opinion risk assessment and monitoring (34–37). Yuan (38) processed the indicators extracted from public opinion information using PCA (Principal Component Analysis) and input the quantitative results into the Support Vector Machine (SVM) model, outputting risk warning estimations as the final results. Sun et al. (39) constructed an Internet public opinion risk early-warning model based on BP neural networks and genetic algorithms. Huang et al. (40) proposed four secondary indicators and 10 tertiary indicators from the dimensions of physical and social attributes. Using 150 earthquake events as case studies, researchers employed an accelerated genetic algorithm to optimize the BP neural network model for measuring the risk levels of earthquake-related Internet public opinion. The aforementioned methods are often labeled ‘black box’ models, as their internal working mechanisms and decision-making processes are hardly interpretable (41). They fail to clearly demonstrate the causal relationships and conditional dependencies between variables, making it impossible to conduct in-depth research into the causes of different levels of Internet public opinion risks. The occurrence of Internet public opinion crises is typically the result of interactions among various risk factors. Identifying key risk factors and the risk paths between them is of great significance for preventing the onset of such crises.

Therefore, the public opinion risk assessment method that integrates association rule mining and BN, by constructing a causal association network of risk factors, helps to address the limitations of machine learning algorithms in interpreting the risk transmission mechanism. This approach can identify the key issues in the risk assessment results more quickly, and adopt more targeted and rapid risk pre-control measures, which is of great significance for achieving precise hierarchical control of public opinion risks.

### 2.2 Bayesian network (BN)

The Internet public opinion triggered by public emergencies exhibits greater complexity and uncertainty. Although the application of machine learning has enhanced the computational capabilities for feature extraction and hotspot detection in public opinion, the integration of machine learning with the inferential abilities of public opinion has become a new research pathway for assessing the current situation of public opinion. In the field of artificial intelligence, BN possesses significant advantages in expressing uncertainties and conducting causal reasoning. As an effective tool for solving complex system problems, it has played a substantial role in numerous domains (42–46). Some scholars have used BN method to research Internet public opinion risk assessment and achieved relevant results. For example, Liu and Wu (47) constructed a BN model for Internet public opinion assessment by determining causal relationships among risk factors through literature and expert knowledge, then integrating objective data via parameter learning. Li et al. (48) utilized the Interpretive Structural Modeling (ISM) to identify causal paths and hierarchical relationships among risk factors of social media network public opinion in emergencies. Based on this, they constructed a risk identification and early warning model for social media network public opinion on emergencies based on BN. Luo and Ma (49) established a BN multi-level fermentation early warning model based on real-world examples of Internet public opinion fermentation, and diagnosed the causes of fermentation using the MPE (Most Probable Explanation) principle. Tian et al. (50) constructed a DBN (Dynamic Bayesian Network) model for early warning scenarios based on the knowledge element model, and trained and tested the model using the EM (Expectation–Maximization) algorithm, elucidating the influencing factors of public opinion crisis warnings under various scenarios.

The above studies often rely on expert subjective experience in constructing the BN topology structure, lacking support from objective laws driven by data-driven approaches, thus exhibiting strong subjectivity. Therefore, this paper proposes a new method to construct the BN topology structure for the Internet public opinion of public emergencies, aiming to address the limitations in prior knowledge.

### 2.3 Association rule mining

Association rule mining, initially proposed by Agrawal in the context of supermarket basket analysis (51), is a method for exploring the potential inter-relationships among itemsets within a database. It has emerged as a prominent research area in data mining and found successful applications across various domains. By analyzing extensive accident data, association rules can uncover associative patterns among uncertain risk factors leading to accidents, aiding in the identification of causal relationship between risk factors and supporting managerial decision-making. Hong et al. (52) employed Apriori algorithm to study the interplay among highway dangerous goods transportation accidents and factors such as driver gender, weather, and routes. Li et al. (53) maked full use of modern information technology to establish a reasonable data-driven coal mine safety risk factor identification model for scientific analysis of accident cases. Based on the extended Apriori algorithm and Markov Chain, Shi et al. (54) analyzed the correlation and transfer between Internet users’ emotion classes and predicted the changing trends of Internet users’emotional states in the early stage of the pandemic. Its application in the field of Internet public opinion is currently relatively scarce. Compared with other fields, the degree of unstructuredness of Internet public opinion data is relatively high. It mostly exists in the form of text, and often requires the integration of natural language processing techniques for text mining, sentiment analysis, etc. Public opinion risk indicators such as the number of comments and reposts, participation levels, emotional polarity are usually difficult to qualitatively process into Boolean - type datasets. Therefore, although the association rule algorithm has certain application potential in the field of Internet public opinion, compared with other fields, its research and practice are still relatively lagging behind.

Compared to parametric methods, association rule mining, a non-parametric machine learning approach, does not rely on any assumptions or prior knowledge, and it is easy to use and provides readily interpretable results. The occurrence of Internet public opinion accidents often involves multiple factors, which are often interrelated. Using association rule mining method mine the hidden association rules between them, has an important role in revealing the risk cause chain of Internet public opinion risk on emergencies. Therefore, this study uses the association rule mining to extract the strong association rules between the risk factors in Internet public opinion risk accident cases, which lays the foundation for further streamlining the risk-causing factors and building the BN topology.

## 3 Methodology

**Figure 1:**
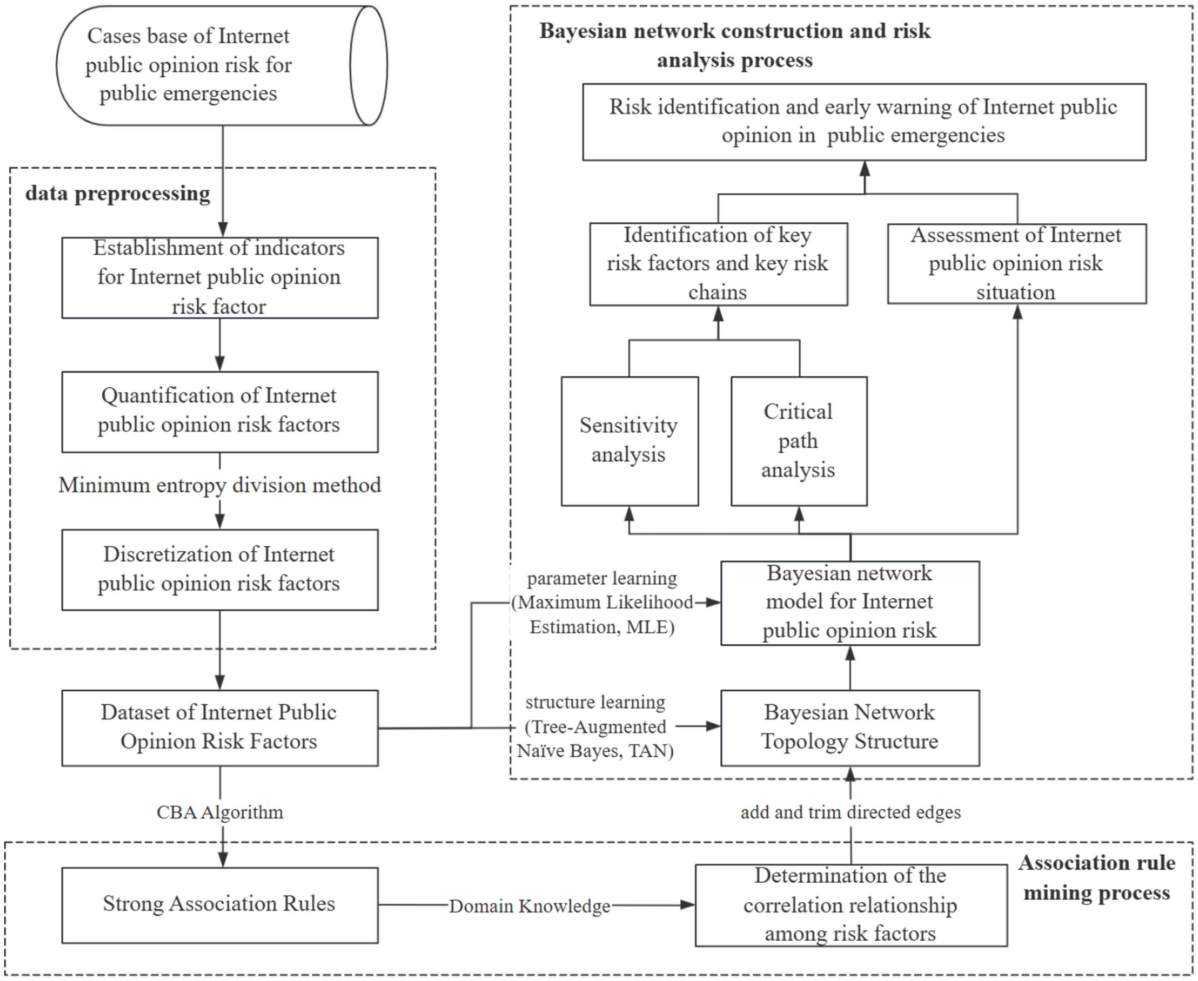
Flowchart for risk identification and assessment of Internet public opinion on public emergencies.

This paper proposes a novel methodology integrating association rule mining with BN for public opinion risk identification and assessment, aiming to enhance the performance of BN evaluation models while simultaneously identifying critical risk factors and pathways under different risk levels. The research framework consists of three core processes (shown Figure 1): data preparation process, association rule mining process, and BN construction and analysis process. Compared with conventional BN applications in public opinion risk analysis, this approach achieves the mining of the association patterns of public opinion risk factors and the systematic deconstruction of multi-level risk transmission chains. By integrating association rule mining for pattern discovery with BN for causal reasoning, it overcomes the limitations of traditional expert-dependent assessments. It provides methodological support that combines computational reliability and decision-making operability for the governance of public opinion.

### 3.1 Establishment of internet public opinion risk factors and data preprocessing

Internet public opinion is a complex evolutionary process of dynamic interaction among multiple entities such as events, netizens, media, and government (55). In different emergency situations, members of online groups will experience varying emotional responses, which drive their distinct information behaviors. This is an important indicator for measuring the development of public opinion and changes in its intensity (56). The public’s trust in the media can influence netizens’ emotional inclinations and levels of attention (57). Based on a review of relevant literature, expert interviews, and the analysis of numerous real cases of Internet public opinion risk in public emergencies, we summarize that the evolution of Internet public opinion is primarily the result of the coupling effect of multiple factors. These factors include sentiment orientation, netizens’ information behavior, public opinion attention, dissemination and diffusion patterns, and the intrinsic attributes of the events themselves. Thus, during the assessment of Internet public opinion risk, it is essential to fully account for the intrinsic interaction mechanisms among multiple factors, thereby enabling more comprehensive and accurate dynamic risk assessments and early warnings for public opinion development trends.

The Internet public opinion risk factors are as shown in Table 1. On this basis, complete data collection and quantification, and apply the minimum entropy partition method to discretize continuous numerical indicator data.

**Table 1:** Internet public opinion risk factor identification.

Risk category	Risk factor	Factor explanation	Reference source
Sentiment Orientation of Public Opinion	Governmental Sentiment Tendency (*A*_1_)	The government’s subjective emotional attitude toward emergencies in general can be classified into three categories: positive, neutral, and negative.	(62–64)
	Media Sentiment Tendency (*A*_2_)	The media’s subjective emotional attitude toward emergencies in general can be classified into three categories: positive, neutral, and negative.	
	Netizen Sentiment Tendency (*A*_3_)	The netizen’s subjective emotional attitude toward emergencies in general can be classified into three categories: positive, neutral, and negative.	
	Netizen Sentiment Polarization (*A*_4_)	Whether there is a consensus of extreme emotions among netizens toward emergencies.	
Online Information Behavior of Netizens	Like-to-View Ratio (*B*_1_)	The ratio of user likes to views is categorized into three levels: low, medium, and high.	(65, 66)
	Comment-to-View Ratio (*B*_2_)	The ratio of user comments to views is categorized into three levels: low, medium, and high.	
	Repost-to-View Ratio (*B*_3_)	The ratio of user reposts to views is categorized into three levels: low, medium, and high.	
Public Attention Level to Opinion	Governmental Authority Level (*C*_1_)	The administrative levels of government agencies are classified into: central government, provincial government, and municipal and county-level governments.	(67–70)
	Government Participation Level (*C*_2_)	The level of government agencies’ involvement in public opinion regarding emergencies, through the issuance of documents, taking into comprehensive consideration the number of documents issued by government agencies and the hierarchical level of the issuing agencies.	
	Media Authority Level (*C*_3_)	The hierarchy of social media is classified into central-level media and non-central-level media.	
	Media Participation Level (*C*_4_)	The level of social media’s involvement in public opinion regarding emergencies, through the issuance of posts, taking into comprehensive consideration the number of posts issued by social media and the hierarchical level of the issuing media platforms.	
	Netizen Participation Level (*C*_5_)	The level of netizens’ involvement in public opinion regarding emergencies, through the issuance of posts, taking into comprehensive consideration both the number of posts issued by netizens and the influence of the posters.	
	Government Response Speed (*C_6_*)	It refers to the timeliness of the government’s response and handling of the public opinion situation of emergency incidents.	
Dissemination and Diffusion Extent of Public Opinion	Cyberspace Public Sentiment Dispersion (*D*_1_)	The diffusion and dissemination of public opinion information in cyberspace, with a focus on the geographical distribution of netizens who are paying attention to the emergency event.	(71, 72)
	Forms of Public Opinion Dissemination (*D*_2_)	The media for publishing information related to emergencies, including text, images, and videos.	
	Duration to Attain Opinion Climax (*D*_3_)	The time taken for public opinion regarding an emergency to reach its peak from its inception.	
	Duration of Public Opinion (*D_4_*)	The entire cycle during which public opinion persists after the outbreak of an emergency incident.	
Intrinsic Attributes of Public Opinion Events	Public Harmfulness of Emergencies (*E*_1_)	The extent of social harm caused by emergencies.	(47, 73)
	Categories of Emergencies (*E*_2_)	Categorized into: public health, natural disasters, accidental disasters, and social security.	
	Degree of Membership for Similar Events (*E*_3_)	Whether there were similar events occurring before the outbreak of the emergency, and the degree of similarity between the events measured.	
	Degree of Membership for Emergencies (*E*_4_)	The extent to which the event can be considered as an emergency.	

Addressing the characteristic that association rule mining and BN cannot handle continuous values, the minimum entropy partitioning method is applied to complete the data discretization process. In this paper, let the indicator to be discretized be denoted as A , its dataset as I , and the discretization boundaries as T . The class information entropy under the partitioning induced by T is defined as:


$$E(A,T;I)=\frac{|I_{1}|}{|I|}\mathrm{Ent}(I_{1})+\frac{|I_{2}|}{|I|}\mathrm{Ent}(I_{2})$$

Here, $\mathrm{Ent}(I_{i}),i=1,2$ is the information entropy of the dataset, defined as:


$$\mathrm{Ent}(I)=-\sum_{i=1}^{n}P(x_{i})\log_{2}P(x_{i})$$

In the formula, n denotes the number of categories after the discretization of the dataset, and $P(x_{i})$ represents the probability of the i -th category appearing in the indicator dataset. For the indicator A and its dataset I , the partition boundary $T_{\min}$ that minimizes the class information entropy is sought as the binary partition boundary for the current round. The recursion stops if and only if the data partitioning meets the following conditions:


$$\text{Gain}(A,T;I)<\frac{\log_{2}N-1}{N}+\frac{\Delta (A,T;I)}{N}$$

Among, Gain(A,T;I)=Ent(I)−E(A,T;I)

$$\Delta (A,T;I)=\log_{2}(3^{k}-2)-[k\cdot \mathrm{Ent}(I)-k_{1}\cdot \mathrm{Ent}(I_{1})-k_{2}\cdot \mathrm{Ent}(I_{2})]$$

N represents the number of elements in the dataset I , and $k_{i}$ denotes the number of samples in the dataset $I_{i}$ . At this point, n−1 partition boundaries for discretizing the continuous data are obtained.

### 3.2 Association rule mining process

Based on the collection and processing of public opinion case data, association rule mining is applied to extract strong correlation relationships among public opinion risk factors, thereby forming the foundation for the construction of the BN topological structure.

This study employs the CBA algorithm to mine association rules among influencing factors. The CBA algorithm is essentially an ensemble mining algorithm, with its core relying on the traditional Apriori algorithm. Compared to Apriori, the CBA algorithm can be applied to rule mining in non-Boolean datasets, making it suitable for deep analysis of risk factors associated with public opinion in emergencies. It comprises two components: a rule generator (CBA-RG) and a classifier (CBA-CB).

The CBA-RG, which is based on the Apriori algorithm, is employed to discover all rule items in a dataset that meet the minimum support requirement. Define the pairs composed of the indicators in the case data set I and their corresponding data as item , and condest as the set of item . Then define the class association rules as follows:


condest→y

y∈Y is one of all the class labels. Referring to the definition of association rules, we refer to condest as the antecedent (LHS) of the class association rule and y as the consequent (RHS). The support count condestCount of condest as the number of samples in dataset I that contain condest . The support count rulesupCount of a rule itemset is defined as the number of samples in dataset I that contain condset and also have a consistent final class label. Based on these definitions, we can then derive the support and confidence of the class association rule condest→y :


$$\text{support}=\frac{\text{rulesupCount}}{|I|}*100\%$$

$$\text{confidence}=\frac{\text{rulesupCount}}{\text{condestCount}}*100\%$$

Based on this, we iterate through the data multiple times to identify rule itemsets that simultaneously satisfy the minimum support and minimum confidence thresholds. Further, we construct a CBA-CB classifier. For a rule r:condest→y , we say that rule r covers sample d if and only if the antecedent condest of r perfectly matches the corresponding attribute values in sample d . Moreover, if the consequent y of r matches the classification of sample d , then rule r is said to correctly classify sample d . For two rules $r_{i}$ and $r_{j}$ , when one of the following conditions is met, it is called $r_{i}>r_{j}$ .

1. The confidence of $r_{i}$ is greater than the confidence of $r_{j}$ ;
2. The confidence of $r_{i}$ is equal to the confidence of $r_{j}$ , but the support of $r_{i}$ is greater than the support of $r_{j}$ ;
3. Both the confidence and support of $r_{i}$ are equal to those of $r_{j}$ , but $r_{i}$ was generated before $r_{j}$ .

Thus, we construct an ordered rule sequence $\langle r_{1},r_{2}\cdots r_{n},\text{default}\_\text{class}\rangle$ as the final classifier. In this sequence, when i>j and $r_{i}>r_{j}$ , default_class is designated as the default class.

**Figure 2:**
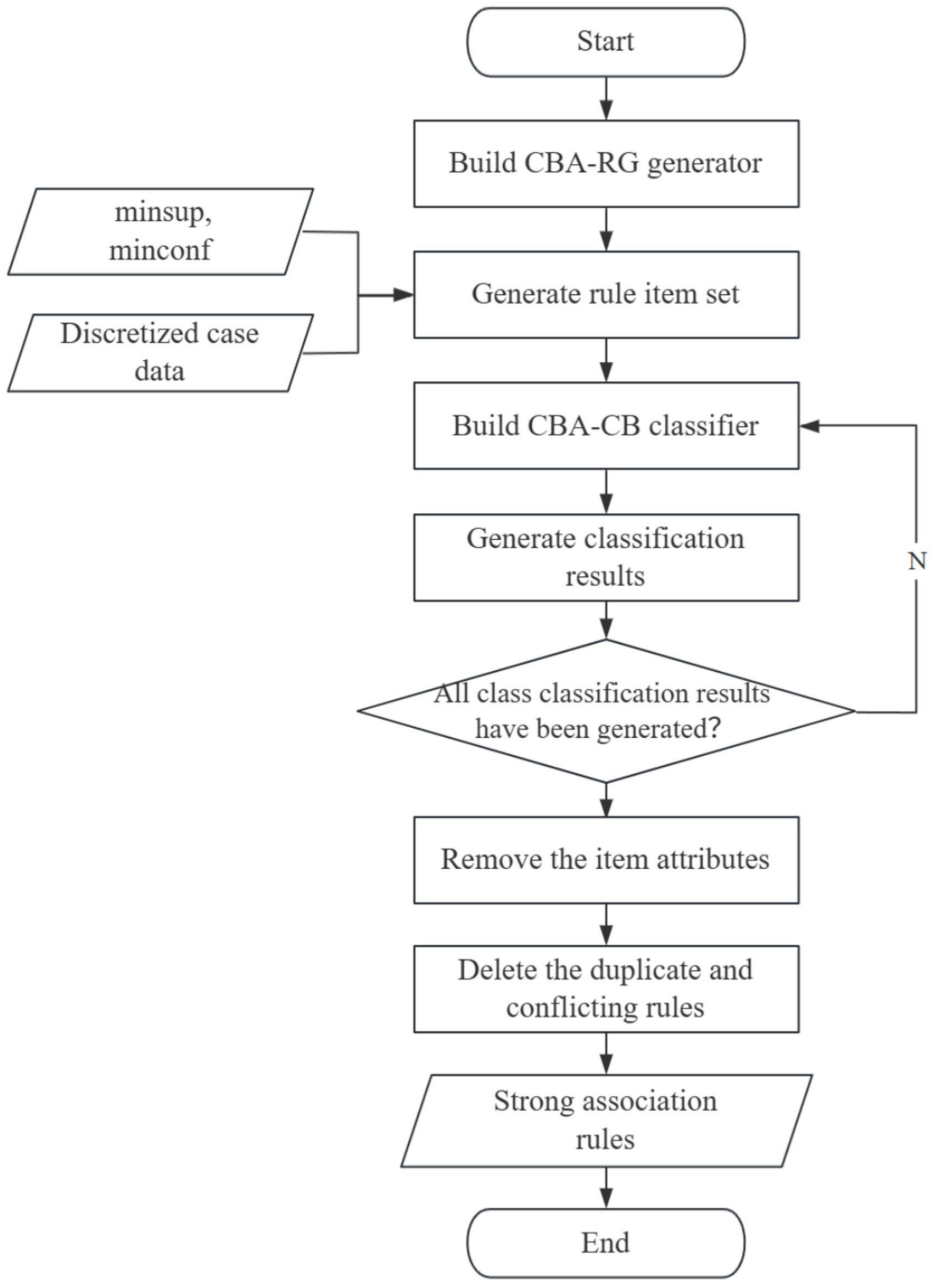
The process of mining strong association rules.

Using the discretized data, take the risk factors and the assessment results of public opinion risk levels as the class in the algorithm, respectively, to repeatedly generate classification results. Then summarize all the classification results as the initial mining results of strong association rules. Among these results, we blur the attribute values of the items in each antecedent (LHS) as well as the class labels of the consequent, that is, without considering the values of the item indicators and the final classification results. It is assumed that the indicators appearing in the antecedent are related to the final class, thus simplifying a class association rule into an ordinary association rule. On this basis, duplicate rules among the obtained association rules are de - duplicated according to the principle of keeping the rule with a higher support. If the supports are the same, the rule with a higher confidence is kept. If both are the same, the rule that was generated first is kept, thus obtaining the final set of strong association rules. The specific process is shown in Figure 2.

### 3.3 Bayesian network (BN) analysis process

Combine the results of strong association rule mining with the BN structure learning to construct the BN topology. Furthermore, through BN parameter learning, establish the BN model that possesses the capability to analyze and assess the risks of public opinion in public emergencies.

1. Apply the Tree-Augmented Naive Bayes (TAN) algorithm for BN structure learning to establish a fundamental BN topology driven by data.
2. Based on the strong association rules of public opinion risk factors, supplement and prune the directed edges in the network topology to obtain the final BN structure.
3. Further, apply the Maximum Likelihood Estimation (MLE) method for BN parameter learning to construct a BN model with the ability to identify and assess public opinion risks.
4. Through the sensitivity analysis and critical path analysis based on this BN model, identify the key influencing factors of Internet public opinion risks in emergencies and the key causal paths of different risk levels.
5. Finally, through the comparative analysis of empirical results, it is demonstrated that the public opinion risk BN assessment model based on strong association rules exhibits better performance.

## 4 Results and analysis

### 4.1 Data collection and preprocessing

This paper select 105 typical Internet public opinion events triggered by public emergencies in recent years as the research objects, which cover various emergency scenarios including public health, accidental disasters, natural disasters, and social security, ensuring the comprehensiveness and universality of the case data. Weibo is the main discourse gathering place for public emergencies, and “Zhiwei Shijian” is a powerful, authoritative, and widely-covered platform for tracking hot events across the entire Internet. The cases data are sourced from Weibo and “Zhiwei shijian.” Retrieve the monthly “Top 10 Influence Index List” from the “Zhiwei Shijian” platform for the period spanning August 2020 to March 2024. The Event Influence Index (EII) is an authoritative metric that evaluates the communication effectiveness of individual events on the Internet, utilizing self-media and online media data from comprehensive web sources. A total of 128 Internet public opinion incidents triggered by emergencies (including social issues, disasters, and illegal activities) are selected, with partial cases shown in Table 2.

**Table 2:** Typical Internet public opinion case events (partial).

Event name	Category	EII	Date
Explosion in Yanjiao Town, Hebei	Society, Explosion	82.5	2024-03-13
Fire at a street-side shop in Yushui District, Xinyu City, Jiangxi	Society, Fire	80.0	2024-01-24
Landslide in Zhenxiong, Yunnan	Society, Natural Disaster	83.2	2024-01-22
Collapse of a gymnasium in Jiamusi	Society	81.0	2023-11-06
2023 Hebei Rainstorm	Society	89.4	2023-07-27
Assault on female patrons at a Tangshan barbecue restaurant	Society, Violent Crime	89.9	2022-06-10
Shanghai citywide lockdown due to resurgence of local COVID-19 cases (March 2022)	Society, Pandemic	100.0	2022-03-01

Next, through web crawling technology, we obtain blog posts related to various events, extract basic information such as forwards, comments, likes, publisher authentication, location, and number of followers for each post, and simultaneously crawl the full text of comments, comment timestamps, and comment posting locations. On the other hand, through manual transcription, we obtain statistical data such as peak event popularity and the time taken to reach the peak popularity. Following the initial data collection phase, events in the lowest 20th percentile of post volume or comment volume are excluded (retaining 105 valid cases after filtering). Adhering to the classification framework outlined in “National Emergency Response Plan for Public Emergencies,” the samples are categorized into four major typologies: public health, accidental disasters, natural disasters, and social security incidents (shown Table 3). From this, we obtain 105 sets of original case data covering the full lifecycle of public emergencies, including both textual and quantitative digital information. After deduplication and cleaning processes, a total of 89,548 blog posts and comment texts related to various events are ultimately obtained.

**Table 3:** The Internet public opinion case data distribution.

Event type	EII	Number of cases
Public health	70.6 ~ 100	37
Natural disaster	75.5 ~ 98.4	30
Accidental disaster	82.5 ~ 94.0	16
Social security	73.3 ~ 89.9	22

The emotional orientation of governments, media, and netizens reflect their attitudes toward the public opinion event, exerting a crucial influence on the outbreak of public opinion risks. Using SnowNLP, we calculate the mean sentiment orientation values of all blog posts and comments released by governments, media, and netizens for each case in a grouped manner. SnowNLP is an effective sentiment analysis tool for Chinese texts, which has advantages in Chinese sentiment analysis and has been widely applied to the research on sentiment analysis of social media texts in the field of public opinion (58, 59). SnowNLP computes a sentiment score ranging between 0 and 1 to quantify textual sentiment intensity, where values closer to 1 signify positive sentiment orientation, while those approaching 0 indicate negative affective orientation (60). At present, some studies adopt a three-segment classification method with boundaries of {0.4, 0.6}, categorizing text sentiment into three types: negative, neutral, and positive (61). With reference to existing research, we selected the division boundaries: {0.4,0.6} . When the sentiment score greater than 0.6, it is labeled as 2, indicating a positive sentiment; when it is less than 0.4, it is labeled as 0, indicating a negative sentiment; otherwise, it is labeled as 1.

Further, for an emergency, we calculate the *Z*-score of the text published by netizens:


$$Z-\text{score}(V)=\frac{V-\bar{V}}{S}$$

V is the text sentient orientation value, $\bar{V}\;$ is the average value of text sentient orientation value released by all netizens of the event, and S is the standard deviation of text sentient orientation value released by all netizens of the event. When ∣Z−score(V)∣>3 , the text is defined as extreme emotional text. When extreme emotional texts in an event account for more than 5% of the total number of texts published by Internet users, it is considered that there is netizen sentiment polarization in the public opinion of the event, and the scale is 1; Otherwise, the scale is 0.

Netizens’ information behaviors provide multifaceted feedback on their degree of concern and importance attached to an event. Calculate the total number of likes, retweets, comments, and views for all texts, including blog posts and comments, in each case. Assign values to the three indicators of netizens’ information behaviors by computing the ratios of total likes, total retweets, and total comments to the total number of views, respectively. The indicators of government authority and the media authority are assigned values based on the highest-level government agency involved in the release of public opinion information and the tier of social media platforms, respectively. The indicator of government participation is obtained by weighted summation of the volume of posts by government agencies and the levels of those posting agencies. Similarly, the indicator of media participation is derived by weighted summation of the volume of posts on social media platforms and the levels of the posting entities. The netizens attention indicator also focuses on the quantity of posts made by netizens and the influence of the posting entities. Here, we assign weights of 2 and 1 to posts by major influencers (so-called “Big *Vs*”) and ordinary users, respectively, and calculate this indicator through weighted summation.

In terms of government response speed, this paper focuses on the time consumed from the occurrence of emergencies to the first implementation of rescue, control, guidance and other measures by government agencies. We select the implementation time of the rescue and control measures first disclosed by the government agencies, and the blog posting time of the government agencies’ first positive response to emergencies, whichever is less time-consuming, as the quantitative result of the government response speed factor. The unit of the factor is hour.

The form of public opinion dissemination is represented by the most complex information dissemination form appearing in the relevant public opinion blog posts. Specifically, if it contains videos, the scale is 2; if it only contains pictures and text, the scale is 1; if it only contains text, the scale is 0. The reference for the time taken to reach the peak of public opinion is based on the trend chart of public opinion popularity for various events on the “Zhiwei Shijian” website, which is defined by the time elapsed from the emergence of public opinion to its peak popularity. Regarding the duration of public opinion, it also refers to the public opinion popularity trend chart of each event on the “Zhiwei Shijian” website. Calculate the time elapsed from the emergence of the public opinion heat of the event to the moment when the heat of the public opinion event drops to 0 for the first time.

The public Harmfulness of Emergencies is classified into four levels: particularly serious, serious, relatively serious, and general, based on the nature of the event, the degree of harm, and the scope of involvement, etc. The lifecycle of Internet public opinion typically does not exceed 7 days. If a similar event occurred within the preceding 7 days, the degree of similarity for that event is scaled as 1; otherwise, it is 0. The risk level of Internet public opinion in public emergency relies on the evaluation of domain experts. The Delphi method is employed to determine the risk level through multiple rounds of anonymous assessments by multiple experts.

Association rule mining and BN are unable to handle continuous data. In this paper, the aforementioned minimum entropy partitioning method is used to discretize various continuous numerical indicators, establishing demarcation boundaries for each metric.

### 4.2 Association rules mining analysis

The fermentation of Internet public opinion is usually caused by the interaction of multiple risk factors. Identifying key risk factors and their key causal paths, and subsequently restraining these factors, play a crucial role in preventing the risks associated with Internet public opinion. Based on the quantitative processing of the collected indicator data, this study employs the classic CBA algorithm to mine the strong association rules between the risk factors of Internet public opinion, laying the foundation for the identification of public opinion key risk factors and the construction of BN topology.

In the process of association rule mining analysis, to obtain optimal mining results, which ensure that a large number of low-quality rules are not generated while important associations between factors are not overlooked, it is necessary to optimize the key parameters of the mining algorithm. Support and Confidence are two important condition variables and the setting of their thresholds directly affects the results of association rule mining. There is currently no optimal practice standard for setting the minimum support and confidence thresholds in the CBA algorithm. Therefore, we adopted a trial-and-error approach to test various combinations of minimum support and minimum confidence, and evaluated the effectiveness of the mining results under each combination integrating expert experience and domain knowledge. Ultimately, after repeated testing, the minimum support threshold was set to 0.1 and the minimum confidence threshold was 0.5. The extremely frequent itemset (shown in Table 4) and 154 strong association rules (partial results shown in Table 5) were obtained by mining. The factors contained in the extremely frequent itemset indicate that they frequently occur in Internet public opinion accident cases and are the key cause of Internet public opinion risk. From Table 4, we can see that it does not include the two risk factors, C6 and D4. We conducted a single-factor significance analysis on these two factors and found that they do not have a significant impact on the evolution results of Internet public opinion. At the same time, after analysis, the excavated strong association rules are in good agreement with the rules and procedures in Internet public opinion risk management practices, reflecting the close correlative connections among these risk factors of Internet public opinion.

**Table 4:** The extremely frequent itemset of risk factors.

Factors included in frequent itemset
*A*_1_, *A*_2_, *A*_3_, *A*_4_, *B*_1_, *B*_2_, *B*_3_, *C*_1_, *C*_2_, *C*_3_, *C*_4_, *C*_5_, *D*_1_, *D*_2_, *D*_3_, *E*_1_, *E*_2_, *E*_3_, *E*_4_

**Table 5:** Strong association rules (partial).

Rules	Support	Confidence
{D2} = > {B3}	0.789	0.714
{B2} = > {B3}	0.789	0.667
{D2} = > {C2}	0.737	0.778
{D2} = > {B2}	0.737	0.769
{C4} = > {A1}	0.737	0.750
{C2} = > {C4}	0.684	1.000
{D2} = > {B1}	0.684	0.929
{C2} = > {B3}	0.684	0.929
{B2} = > {C5}	0.684	0.889
{C2} = > {E4}	0.684	0.750
{D2} = > {C4}	0.684	0.600
{D2} = > {C5}	0.632	1.000
{C4} = > {C3}	0.632	0.833
{C2} = > {B2}	0.632	0.800
{B3} = > {C5}	0.632	0.800
{C2} = > {D2}	0.632	0.750
{C3} = > {A1}	0.632	0.667
{A4} = > {B3}	0.632	0.600
{E4} = > {B1}	0.579	1.000
{E4} = > {B2}	0.579	0.833
{A1} = > {A3}	0.579	0.818
{C4} = > {B3}	0.579	0.800
{E3} = > {B3}	0.579	0.692
{B1} = > {C5}	0.579	0.692
{B2} = > {A4}	0.579	0.667
{B2} = > {A1}	0.579	0.643
{C2} = > {A3}	0.579	0.500
{C2} = > {C5}	0.526	1.000
{E3} = > {C2}	0.526	1.000
{D2} = > {A4}	0.526	0.833

As shown in the first rule in Table 5, the form of public opinion dissemination has a strong correlation with the ratio of retweets, indicating that different forms of communication, such as text, images, videos, etc., play distinct roles in attracting netizens’ attention and stimulating their desire to forward. The dissemination forms that provide richer and more intuitive information are more likely to attract netizens, thereby prompting them to engage in retweeting behavior. Similarly, for the rule {B2} = > {B3}, the comment volume serves as a “signal” of content value for internet users, prompting their tendency to share content that sparks extensive discussions to reduce personal screening costs. Simultaneously, high-frequency commenting activity may foster “group identity,” motivating netizens to participate in topic discussions through forwarding behaviors to acquire social capital. These factors collectively contribute to the phenomenon where deep interactions drive dissemination behaviors. The rule {D2} = > {C2} demonstrates the correlation between public opinion propagation patterns and government engagement levels. The analysis of the reasons is as follows: Compared with pure textual content, multimodal content exhibits characteristics of strong sensory impact and high authenticity, making it particularly conducive to spreading extreme emotional content. This accelerates the formation of public opinion hotspots and hastens sentiment fermentation, thereby compelling governments to shorten decision-making cycles, triggering intervention from higher-level institutions, and potentially even initiating administrative accountability processes.

Due to space constraints, we cannot elaborate on each association rule. Overall, these rules reflect how governmental/media guidance, inherent characteristics of emergencies, and inter-group dissemination patterns collectively shape the public sentiment and cognition. The emotional contagion and cognitive influence among groups further steer the evolution of public opinion, aligning with existing research and empirical observations. Furthermore, by analyzing the strong association rules obtained from mining, we find that there are such reasonable but conflicting situations such as {D2}= > {C2}, {C2}= > {D2} at the same time. Therefore, further processing is required when applying strong association rules to build BN topology.

Grounding on the training of the basic Bayesian network structure, the results of association rule mining on Internet public opinion accident case data are comprehensively used to construct a BN topology.

Naive Bayesian Networks (NBN), Augmented Naive Bayes (ABN), and Tree-Augmented Naive Bayes (TAN) are several commonly used data-driven BN network structure learning algorithms. NBN assumes conditional independence among various nodes in the network. This often does not conform to the actual situation. ABN has problems such as increased complexity, high data requirements, an increased risk of overfitting, and reduced interpretability. TAN is an improvement of NBN. It relaxes the independence assumption of NBN and maintains the robustness and computational convenience of NBN at the same time. Therefore, we use the TAN algorithm to train the basic Bayesian network structure.

After the learning is completed, based on this basic network structure, it is modified by using the results of association rule mining. The antecedent and subsequent terms of the strong association rule are regarded as nodes in the network structure, and the association relationship between the antecedent and subsequent items is regarded as a directed edge. Since there are conflicts between the mined strong association rules and the BN structure obtained through structure learning, such as the simultaneous existence of {D2}= > {C2} and {C2}= > {D2} in the rules, or the strong association rules being contrary to the directed edges obtained from structure learning, or the formation of loops in the network after adding directed edges according to the association rules, it is necessary to correct these conflict issues. To obtain a more accurate network structure, in cases of conflicts, we follow the following rules to rectify the conflict issues:

1. When a strong association rule conflicts with the basic structure of the BN, delete the conflicting directed edge in the basic structure and retain the edge of the strong association rule. For example, in the BN structure obtained based on the TAN algorithm, there is a conflict between edge A3 → C2 and the rule {C2}= > {A3}. We delete edge A3 → C2 and redraw edge C2 → A3.
2. When there is a conflict between strong association rules, retain the rule with a higher Support value. If the Support values of the conflicting rules are the same, retain the rule with a higher confidence value. For example, the support value of rule {D2}= > {C2} is 0.737, and the support value of rule {C2}= > {D2} is 0.632. Therefore, the edge corresponding to {D2} = > {C2} is retained.

We found that among the mined strong association rules, there are no rules related to nodes C6 and D4. At the same time, combined with the mining results of the maximum frequent itemset in Table 4, it shows that the correlations between these two factors and other factors in this BN are weak.

For the factor of the government’s response speed (C6), we believe there are two reasons. Firstly, the government’s response speed is not strongly correlated with the response effect. If government agencies only pursue a quick statement but are perfunctory in form, it is likely to exacerbate public distrust. And we believe that the response effect can be better reflected indirectly through factors C1 and C2. Secondly, the government’s credibility is a prerequisite for the impact of the government’s response on the development of public opinion. Limited by their credibility levels, the effectiveness of the public opinion response measures of various government agencies will also be impaired, further weakening the role of the response speed in the risk prevention and control process.

For the factor of the duration of public opinion (D4), on the one hand, a long duration of public opinion does not necessarily lead to risk escalation. For example, in the “Tangshan Barbecue Restaurant Assault Case,” the authorities gradually resolved public doubts by continuously reporting the progress of the case and disclosing the judicial procedures. Although the time span was long, the risk was controllable. On the other hand, in the all-media era, the information update speed is extremely fast, and the duration of a single public opinion is compressed, and the public’s attention shifts rapidly. For example, the massive information flow during the sudden outbreak of the epidemic shortens the life cycle of most public opinions, breaking the linear correlation between the duration and the risk level. These reasons jointly eliminate the correlation between the duration of public opinion and the development of public opinion risks.

**Figure 3:**
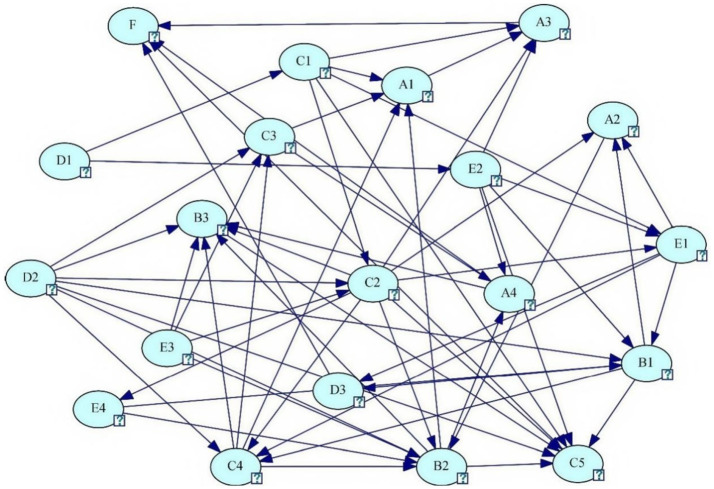
BN topology of Internet public opinion accident.

In conclusion, we removed nodes C6 and D4 from the network and obtained the final BN topology of Internet public opinion, as shown in Figure 3, where node F represents the Internet public opinion event.

### 4.3 Bayesian network analysis

The purpose of Internet public opinion BN construction is to clarify the key risk factors, risk paths and risk levels of Internet public opinion through quantitative analysis, thereby proposing more targeted public opinion risk prevention strategies. Based on the BN topology constructed above, we completed the parameter learning of the BN using the MLE method, and further analyzed it. The training data used for parameter learning also came from the public opinion case data after discretization processing. This study analyzed and summarized the key risk factors of Internet public opinion through sensitivity analysis and critical path analysis.

#### 4.3.1 Sensitivity analysis

**Figure 4:**
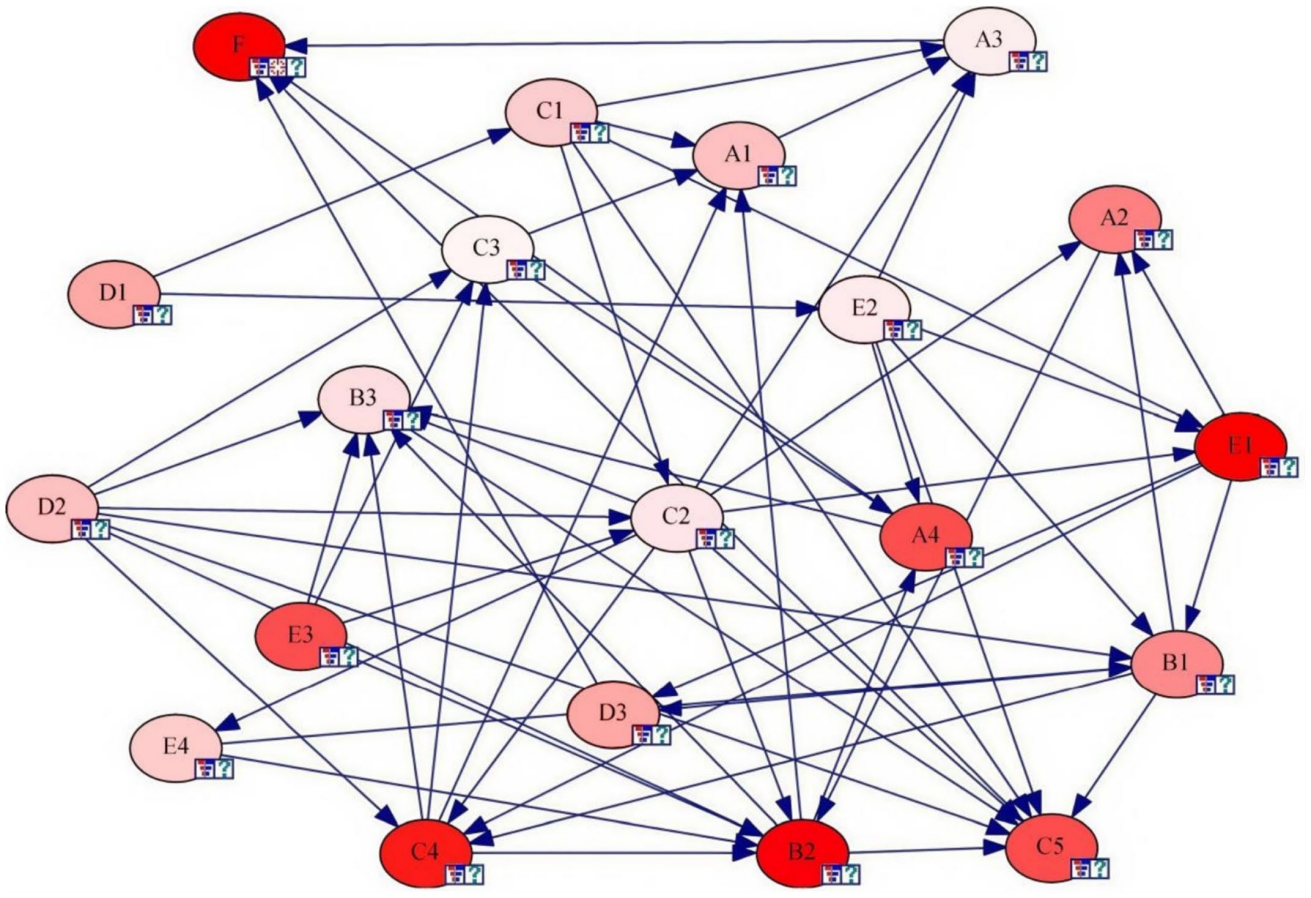
Sensitivity analysis results.

**Figure 5:**
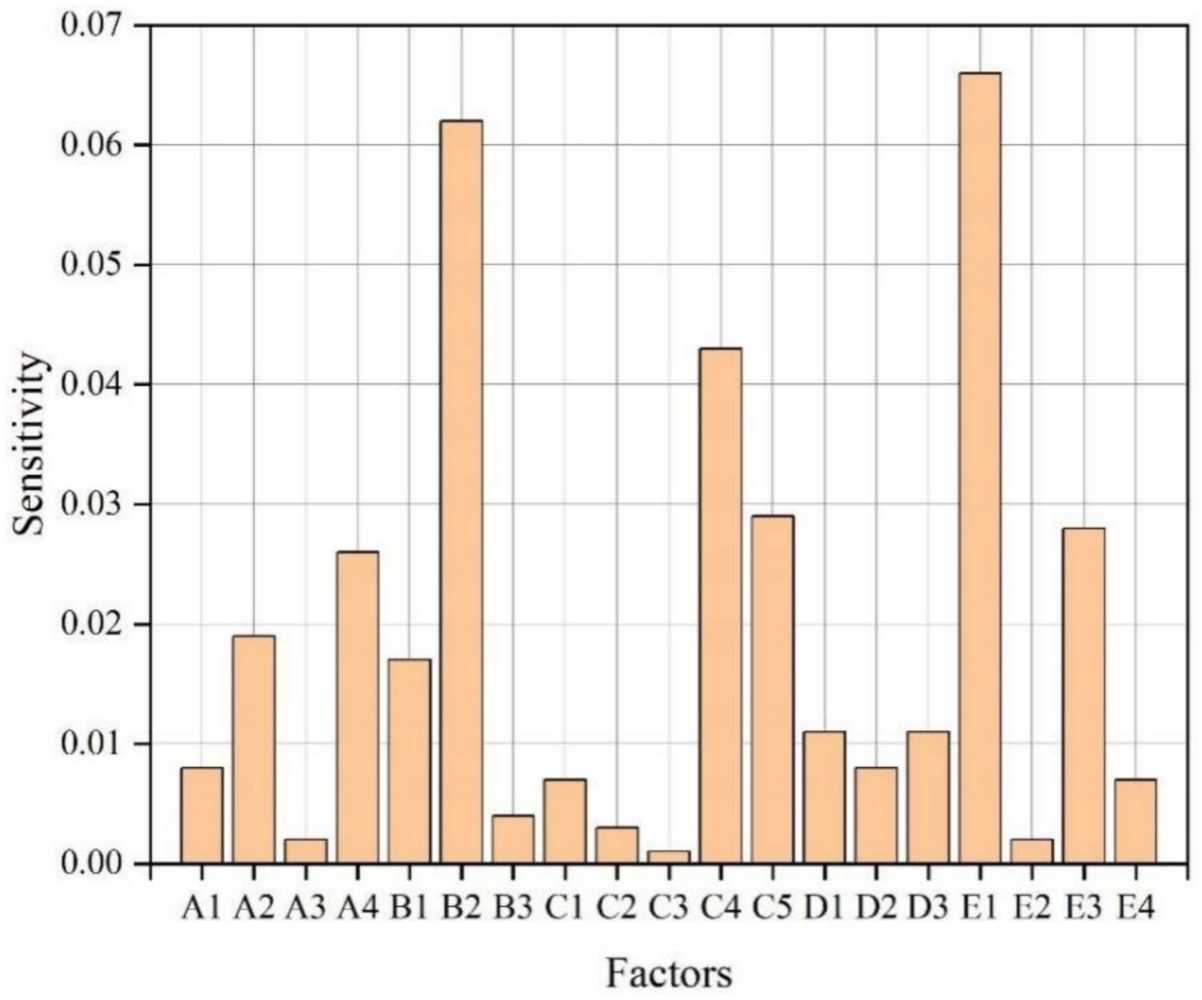
The calculated sensitivity of each node.

Sensitivity analysis is a method widely used to study the influence degree of uncertain factors on key variables in the system. This study employed GENIE 2.0 to analyze the sensitivity of BN node elements, thereby identifying the sensitive factors affecting the Internet public opinion situation and conducting key control of high-sensitivity risk factors, thus clarifying the key direction for managing and controlling Internet public opinion risks. The sensitivity analysis results were shown in Figures 4, 5. Those nodes marked with dark color in Figure 4 are the sensitivity factors affecting Internet public opinion situation. The darker the color, the higher the sensitivity. Figure 5 shown the calculated sensitivity of each node. Combining Figures 4, 5, 10 high sensitivity risk factors were identified: A2, A4, B1, B2, C4, C5, D1, D3, E1, E3. It can be seen, the cause mechanism of Internet public opinion risk is complex, with a large number of sensitive factors. Among these, the higher the sensitivity, the greater the impact on the evolution of public opinion. Relevant regulatory agencies and organizations should focus on monitoring highly sensitive factors in the prevention of public opinion, which is also an effective way to reduce Internet public opinion incidents and improve the emergency management level of major emergencies.

#### 4.3.2 Critical path analysis

**Figure 6:**
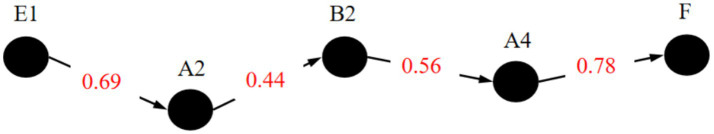
Critical path for high risk.

The research intended to analyze the key occurrence paths of public opinion accidents with different severity respectively, which divided the accidents into low-risk accidents, medium risk accidents and high-risk accidents for analysis. Set the probability of the “high risk” state of the Risk Level node (F) independently to 100% and calculate the joint probability result. It is found that the parent node with the maximum posterior probability of node F is Netizen Sentiment Polarization (A4). At this time, the probability that the state of node A4 takes the value of 1 is 78%. This indicates that the existence of netizen sentiment polarization is the factor with the highest probability of triggering high-level Internet public opinion risks. Continue to set the probability of state 1 of node A4 independently to 100% and conduct reasoning. The parent node with the maximum posterior probability of A4 is obtained as Comment-to-View Ratio (B2). At this time, the probability that node B2 takes state 2 is 56%, and the probability that it takes state 3 is 37%. This shows that a high level of Comment-to-View Ratio is the factor with the highest probability of leading to netizen sentiment polarization. Continue to use B2 as the evidence node. The parent node with the maximum posterior probability of node B2 is Media Sentiment Tendency (A2). When the probability of state 2 of node B2 is set to 100% continuously, the probability that node A2 is in state 1 is 44%, and the probability that it is in state 2 is 30%. This indicates that the negative emotional tendency of the media is the most likely cause of large-scale participation of netizens in event discussions. Repeat the reasoning process until reaching the root node E1. At this time, the key causal path of high-level public opinion risks is obtained, as shown in Figure 6. This path includes four factors: E1, A2, B2, and A4.

**Figure 7:**
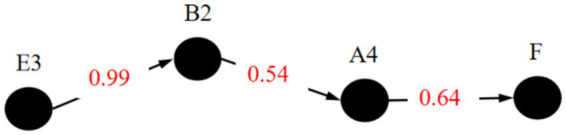
Critical path for medium risk.

**Figure 8:**
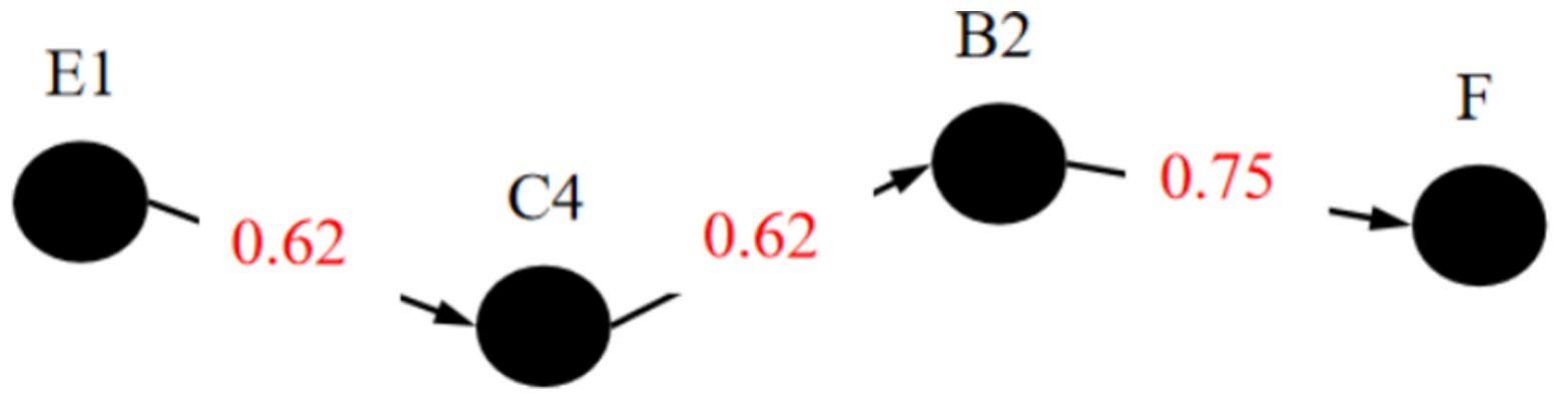
Critical path for low risk.

Similarly, the critical path of Internet public opinion accidents of medium risk and low-risk levels were obtained, as shown in Figures 7, 8. The nodes on the critical path are called the key risk factors causing accidents. It can be seen from Figures 6–8 that there are slight differences in the critical path of different levels of accidents. However, regardless of the level of public opinion risk, by focusing on guiding and controlling online users’ commentary behaviors, particularly those that have the potential to incite emotional polarization among netizens, we can effectively cut off the primary triggers and pathways of diffusion for public opinion risks, thereby preventing the occurrence of public opinion crises.

In the context of emergencies, the widespread distribution of Internet public opinion, coupled with the amplified dissemination effect of the media, particularly its emotional inclination and high participation, significantly heighten internet users’ attention and commentary activity. The media’s emotional orientation shapes public sentiment, while its in-depth involvement accelerates information dissemination and the deepening of public opinion. Simultaneously, the public harmfulness of the event and the perception of historical similarity are key driving factors for the escalation of public opinion risks. The degree of harm directly stimulates public sensitivity, facilitating the spread of public opinion; whereas similar events trigger a “resonance” mechanism, exacerbating the complexity and risk propagation of internet public opinion. Integrating the key factors of three different levels of accidents, the overall key factors of Internet public opinion during public emergencies are: A2, A4, B2, C4, E1, E3.

Integrated the results of sensitivity analysis and critical path analysis, it can be observed that the outbreak of Internet public opinion is significantly influenced by both the inherent characteristics of the emergency itself and the modes of public opinion dissemination. The greater the public harm caused by the emergency, the more likely it is to attract public attention. Furthermore, if similar incidents have already been exposed to the public’s view before the occurrence of the emergency, it may have a promoting or inhibiting effect on the public’s attention to them. However, as factors related to the event’s inherent characteristics, although exert a crucial influence on the outbreak of public opinion risks, they usually cannot serve as entry points for Internet public opinion risk prevention and control. More emphasis should be placed on controlling the dissemination process of public opinion. In the context where streaming media dominates a substantial portion of the public’s fragmented time, plain text alone is becoming increasingly inadequate in attracting deep reading. Instead, it necessitates sufficiently intuitive and diverse modes of dissemination to capture adequate public attention. It is evident that controlling the outflow of multimedia information related to an event serves as a crucial approach to mitigating the risk of public opinion. On the other hand, considering the relationship between netizens’ commenting behavior and the level of public opinion risk, often simple and crude information control measures such as banning comments or closing comment sections are also effective ways to control the fermentation of Internet public opinion.

### 4.4 Assessment of internet public opinion situation

The BN model based on the strong association rules among Internet public opinion risk factors proposed in this paper can effectively evaluate and predict the risk situation of Internet public opinion.

**Figure 9:**
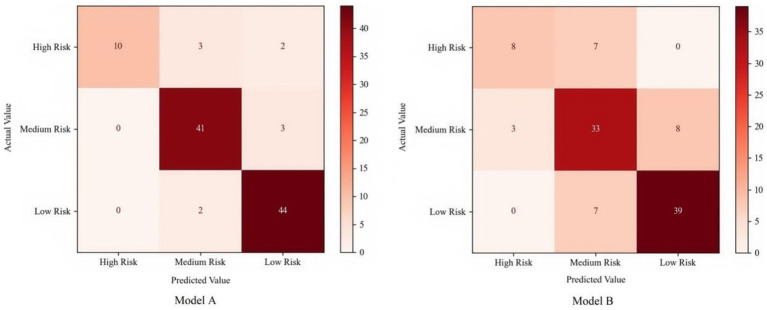
The comparison of the evaluation results of the two models.

Based on the previously constructed BN topology of public opinion, the 5-fold cross-validation method is adopted to train and test the model, so as to verify the accuracy of the BN model combined with association rule mining in evaluating the risk level of public opinion. Set the sample size in the training set to 80% and the sample size in the test set to 20%. In the first simulation experiment, events 1 to 21 are selected as the test set, and the remaining samples form the training set. In the second simulation experiment, events 22 to 42 are selected as the test set, and the remaining samples are used as the training set. This pattern is followed for subsequent simulation experiments. For comparative analysis, a public opinion risk assessment BN model (B) trained using a traditional BN topology structure based on expert experience was adopted as the test control group. The 5-fold cross-validation is carried out on the BN evaluation model (A) based on association rules and the evaluation model (B) respectively. The comparison of the test and evaluation results of the two models is shown in Figure 9.

The evaluation test results shown that for Model A, among the 105 groups of test samples, there were a total of 10 groups of samples whose evaluation results deviate from the actual results. Specifically, these comprised 5 high-risk samples with underestimated risk levels (3 misclassified as medium-risk and 2 as low-risk categories, accounting for 4.76% of the total sample), 3 medium-risk samples with underestimated risk levels (all erroneously categorized as low-risk, accounting for 2.86% of the total sample), and 2 low-risk samples with overestimated risk levels (both incorrectly elevated to medium-risk, accounting for 1.9% of the total sample). This indicates that Model A has the problem of underestimating the risk level to a certain extent, and this problem has a particularly significant impact on high-risk level sample cases. In response to this problem, we believe that it is because in the original 105 groups of case data, the proportion of high-risk level cases is too small, as shown in Table 6. The limited amount of data is insufficient to fully reflect the potential relationship between risk factors and the high-risk level. This requires further collection of high-risk level cases to optimize the public opinion case library.

**Table 6:** Statistics on the distribution of case risk levels.

Risk level	Number of cases	Proportion of the total number of cases (%)
High-risk	15	14.29%
Medium-risk	44	41.9%
Low-risk	46	43.81%

In contrast, Model B had incorrect evaluation results for 25 cases in the same test samples. We found that compared with the Bayesian network topology structure of Model A, it is obvious that the number of directed edges in the topology structure of Model B is significantly reduced, which indicates that Model B did not fully explore the causal relationships between factors and between factors and risk levels, resulting in a less effective risk level evaluation performance compared to Model A. Overall, compared to Model B, the Bayesian network evaluation model based on association rules constructed in this paper achieves an accuracy rate of 90.5% in assessing the risk situation of Internet public opinion. This thus proves the effectiveness of this model in the application of evaluating the level of Internet public opinion situation, and it can be applied in the practical application of risk assessment of Internet public opinion in emergencies.

**Figure 10:**
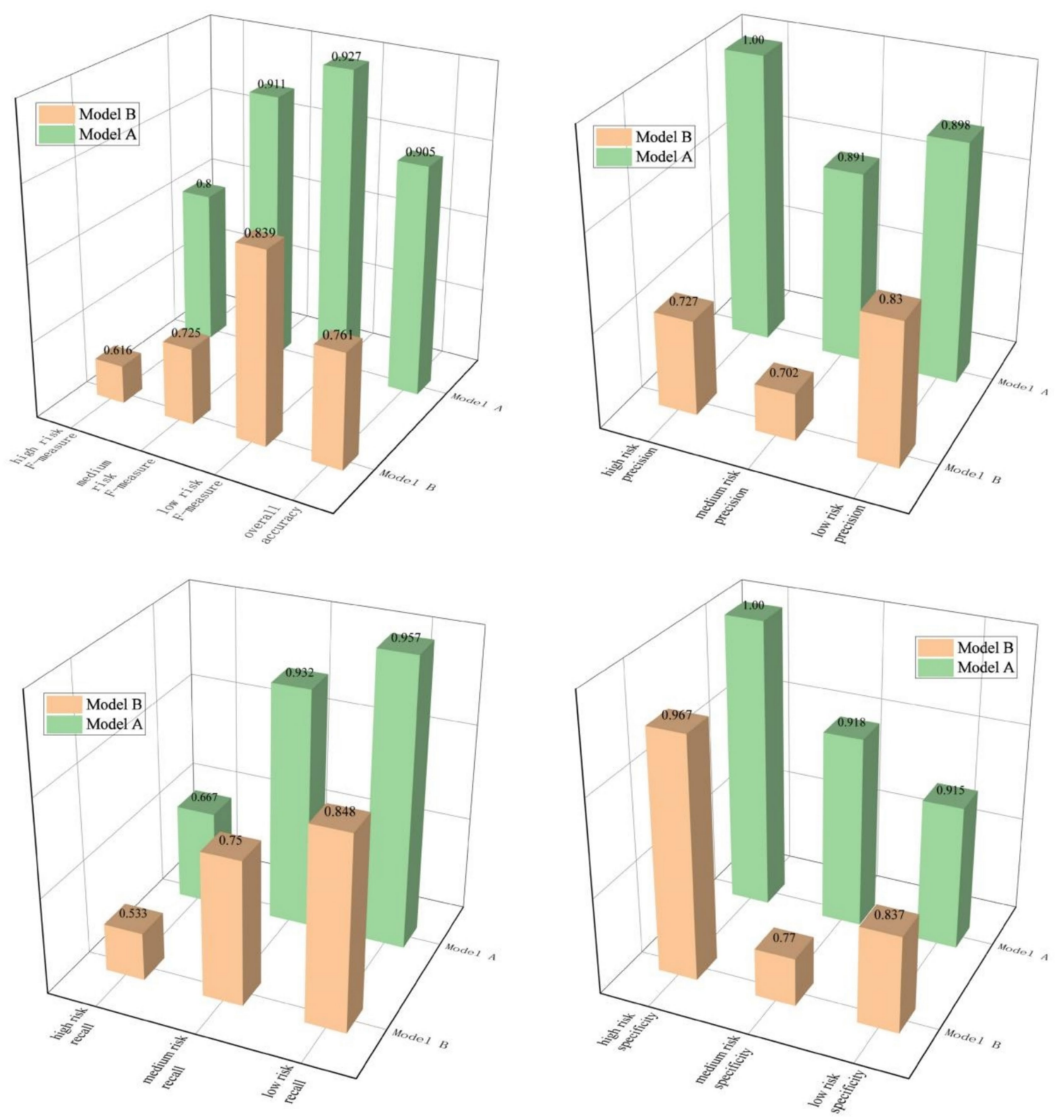
The comparison of the performance of both models.

To further evaluate the performance of both models, this study employs precision, recall, specificity, accuracy, and F-measure as evaluation metrics. These indices are calculated based on the confusion matrix presented in Table 7, with computational results depicted in Figure 10.

**Table 7:** Confusion matrix.

	Predicted High-risk	Predicted Medium-risk	Predicted Low-risk
Actual High-risk	$T_{\text{high}}$	$F_{\text{medium}}$	$F_{\mathrm{low}}$
Actual Medium-risk	$F_{\text{high}}$	$T_{\text{medium}}$	$F_{\mathrm{low}}$
Actual Low-risk	$F_{\text{high}}$	$F_{\text{medium}}$	$T_{\mathrm{low}}$

The experimental results show that, compared with Model B, the Bayesian network enhanced by CBA has increased the accuracy and F-measure scores at three risk classification levels by 14.4, 18.4, 18.6 and 8.8%, respectively. Its performance has obvious overall advantages over Model B. Furthermore, government agencies expect to reduce the missed judgments of higher-level public opinion risks. This is because the missed judgments of higher-level risks will directly expand the risk exposure. If the higher-level public opinion risks cannot be identified in a timely manner, it will lead to an increased probability of the outbreak of public opinion crises, causing direct economic losses and impacts on social stability. From this perspective, the model is required to have good Recall performance when conducting medium and high-level risk assessments. The experimental results show that, in terms of this indicator, Model A has increased by 18.2 and 13.4%, respectively, compared with Model B. On the other hand, it is necessary for government agencies to prevent excessive resources from being invested in frequently responding to misjudged low-level risks by reducing the misjudgments of low-level risks. In this way, sufficient resources can be released for strengthening the continuous and dynamic prevention and control of higher-level risks. This requires the model to have good Precision and Specificity. Compared with Model B, Model A has increased Precision and Specificity by 6.8 and 7.8%, respectively, in the assessment of low-level risks. In fact, from the experimental results, for the three risk levels, Model A outperforms Model B in all terms of precision, recall, specificity, accuracy, and F-measure.

These findings substantiate that the proposed methodology significantly enhances public opinion risk assessment precision, demonstrating closer alignment with actual observations through its advanced pattern recognition capabilities. This methodological advancement shows particular efficacy in multi-level risk evaluation, with measurable practical utility in public emergency internet opinion evaluation scenarios.

## 5 Discussion

### 5.1 Perspective of methodology

Identifying and assessing the underlying causes and paths of the fermentation of Internet public opinion on public emergencies, is the prerequisites and foundations for early warning and formulating intervention measures. Cases of Internet public opinions in public emergencies are highly valuable for extracting the key factors and critical paths that may trigger public opinion crises. Currently, there is a lack of mining of online intelligence knowledge about public emergencies, as well as a lack of collaborative research on identifying key risk factors and assessing risk situations through knowledge learning from prior Internet public opinion cases. Therefore, starting from collecting case data, this research creatively combines association rule mining with BN to conduct a systematic analysis of the risks of Internet public opinion on public emergencies.

This study proposes a BN model optimized with the CBA algorithm for Internet public opinion risk identification and assessment during emergencies, aiming to achieve key factors, paths analysis and risk situations evaluation. As an effective white-box analytical approach, BN enables the integration of data-driven insights and prior knowledge during model construction. However, the conventional BN model construction method has the issue of network structural distortion, so we introduce association rule mining to enhance the process of topological construction. This enhancement facilitates comprehensive extraction of causal relationships among risk factors while ensuring interpretability. Furthermore, we design an improved scheme based on the CBA algorithm to overcome the limitations of traditional algorithms in handling non-Boolean data association mining.

We established the data-driven BN model by utilizing the strong correlation of risk factors, optimized the prior knowledge base for BN topology learning, and improved the accuracy and interpretability of the structure. Compared with expert experience-dependent model, this approach significantly reduces reliance on human subjective experience while constructing complex and interpretable model through objective data analysis. The experimental results also show that the model established by this method has better accuracy, providing reliable decision-making support for emergency management of social media public opinion during crises.

### 5.2 Perspective of content

The evolution of Internet public opinion risks in emergencies is a systematic process characterized by multifactorial interactions that ultimately exert direct or indirect impacts on the occurrence of risk. Empirical analyses through association rule mining and BN reveal that event characteristics, coupled with subjects’ emotional tendencies, behavioral patterns, and participation level, constitute primary determinants of risk evolution, while media and netizens are the main bodies that have a key impact on the evolution of public opinion risk. Notably, emotional guidance from the media can prompt netizens in emergency situations to generate extreme emotions that can trigger high physiological impulses. When extreme emotions become the dominant emotions of netizens, they will have a significant triggering effect on the outbreak of public opinion risks. Meanwhile, the characteristic of the event itself is the lowest level factor of public opinion risk outbreak. High risk events and unconventional events will quickly attract the attention of media institutions, and through the power of the media, affect the emotions of netizens, thereby promoting the outbreak of risks. In the above process, the guidance of the media and the accumulation of netizens’ emotions are reflected through the participation of netizens’ main comments. Throughout the process, media orientation and emotional accumulation can be reflected through quantitative measurement of user engagement in comment interactions.

The fundamental approach to mitigating the risks of Internet public opinion in emergency situations lies in tracing the origin of the incident while giving priority to identifying sensitive information on social media platforms. This process necessitates the integration of heterogeneous informational structures through multi-modal information fusion techniques, enabling intelligent mining, sentiment monitoring and high-risk individual tracking. The implementation of effective process control measures facilitates timely disclosure of event-related information during opinion eruption phases. Strategically leveraging positive emotional vectors to mitigate negative sentiment tendencies constitutes a critical mechanism for effectively dissipating and guiding collective public affect, thereby achieving dynamic emotional regulation within networked populations.

The social media cyberspace represented by Weibo inherently exhibits an ecological nature of information dissemination, permeated with dynamic fluctuating factors. In this context, it is crucial to conduct a multi-dimensional analysis of the evolving state of public opinion risks during emergencies by identifying the risk influencing factors and paths and establishing a risk assessment model. Meanwhile, based on the risk assessment, an early-warning mechanism for network public opinion risks in emergency situations should be implemented, which constitutes pre-event control measures to cope with potential public opinion crises.

### 5.3 Implications

**Figure 11:**
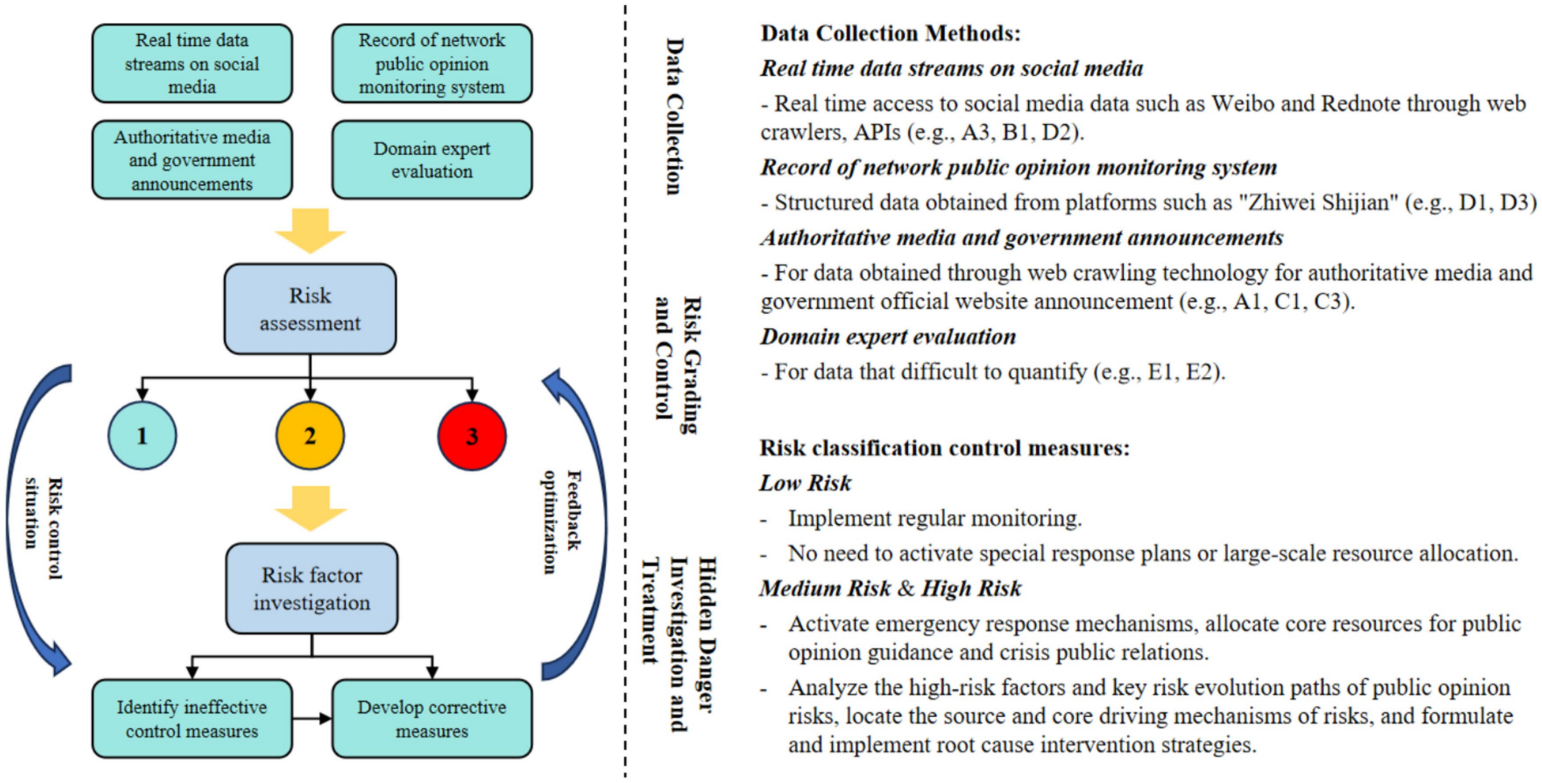
Risk control process.

Our investigation delves into the interdisciplinary nexus of sociology, communication studies, and computer science. As a fundamental component of risk management research, this work proposes an effective methodology for association rule mining in non-Boolean structured data, enhancing the technical adaptability to diverse research scenarios. Building upon this advancement, we construct a data-driven analytical framework through the integration of association rule mining with BN. This framework provides a highly interpretable modeling approach for public opinion risk analysis and assessment, effectively reducing the problem of network structural distortion during the construction process of traditional BN. The proposed methodology establishes an innovative pathway for identifying causes and paths of risk in public opinion, while enabling provide precise and detailed prediction results regarding impending public opinion crises. By delving deeply into the causes of risks and comprehensively perceiving the evolution of risks, a targeted paradigm for preventing public opinion risks can be established. Specifically, with the practice of Internet public opinion risk management and control as the foothold, a dual prevention system for Internet public opinion risk, consisting of risk grading and control as well as hidden danger investigation and treatment, can be established, as illustrated in Figure 11.

As can be seen in Figure 11, the accurate acquisition of high-quality, multi-source heterogeneous data is a prerequisite for ensuring risk assessment. In practical applications, constructing a dataset that comprehensively reflects the public opinion ecosystem relies on systematic development and accumulation. In the stage of risk grading and control, by integrating multi-source data, public opinion risks are identified and assessed based on the analytical framework proposed in this study, and the public opinion risk levels are classified into three tiers: low, medium, and high. The higher the level, the greater the possibility and scale of the current event further triggering a public opinion crisis. In practical risk management scenarios, not all identified risk signals require immediate intervention. For different assessment levels, differentiated governance plans should be formulated. When the assessment result is “low” risk level, the current issue can be considered to be within the scope of regular control. Management agencies mainly conduct routine monitoring, and there is no need to immediately activate special response plans or large-scale resource allocation. When the risk level increases, in addition to immediately activating the emergency response mechanism and allocating core resources for public opinion guidance and crisis public relations, the interpretable analysis results of this study framework can be used to reveal the high-risk triggers and key risk evolution paths of public opinion risks. This enables precise identification of the source of risks, thereby formulating and implementing root-cause intervention strategies to effectively block the risk escalation chain. Furthermore, in the stage of hidden danger investigation and treatment, by dynamically tracking the content and direction of public opinion evolution, the hidden dangers in public opinion risk management and control can be analyzed, and improvement measures can be formulated. Through identifying and addressing gaps, the effectiveness of risk management and control can be further ensured.

In general, the core of Internet public opinion risk pre-control lies in the accurate matching of risk evolution stages and intervention timing. Therefore, the top priority for preventing the outbreak of public opinion risks is to identify key risk factors, establish risk assessment models, conduct multi-dimensional analysis of all elements involved in the evolution of Internet public opinion risks in public emergencies, and implement early warning of Internet public opinion risk situations in the context of public emergencies based on risk assessment.

## 6 Conclusion

Based on the mining and analysis of correlations among public opinion risk factors, this paper constructed a BN model based on strong association rules. This model is designed to analyze the underlying causes of public opinion risks and conduct grade evaluations, thereby addressing the limitations of machine learning algorithms in risk assessment—specifically, their inability to identify risk paths and insufficient interpretability. Constructing a BN structure for Internet public opinion based on strong association rules among risk factors is a feasible and effective method. Based on the 154 strong association rules mined using the CBA algorithm, and combined with BN structure learning, a BN model for Internet public opinion with 19 factor nodes was established. The design of this model fully draws on the experiences and lessons of traditional models to minimize the impact of subjective factors. On this basis, combined with sensitivity analysis and critical path analysis, five key risk factors affecting public opinion incidents and the corresponding critical paths for different risk levels were extracted, providing valuable decision support for determining intervention mechanisms. Finally, through the comparative analysis of the evaluation results, the BN evaluation model trained using the BN topology based on strong association rules has a better performance in risk level assessment, with an accuracy rate of 90.5%. This provides an innovative and feasible approach for the study of Internet public opinion risk assessment in public emergencies.

There are still some limitations in this paper. The single data structure may introduce data bias, failing to comprehensively reflect the diversity and complexity of the research object. Static BN structure assumes time invariance, neglecting the dynamic evolution of risk propagation. Dynamic Bayesian Network (DBN) explicitly incorporate temporal dependencies by integrating time-series data into the modeling framework, thereby enabling more precise characterization of risk dynamics and real-time prediction of risk evolution trajectories. Future research could focus on multi-modal data fusion, integrating heterogeneous data processing techniques with DBN to construct a dynamic multi-dimensional assessment framework. This would enhance the adaptability of public opinion risk identification in the context of data diversity and socio-contextual complexity. For sparse-data scenarios involving rare-event risks, hybrid architectures can be developed to strategically integrate domain expertise with data-driven pattern discovery, thereby achieving human-AI collaborative optimization.

## Data Availability

The raw data supporting the conclusions of this article will be made available by the authors, without undue reservation.
